# Comparison of the outcomes of EMS vs. Non-EMS transport of patients with ST-segment elevation myocardial infarction (STEMI) in Southern Iran: a population-based study

**DOI:** 10.1186/s12873-022-00603-x

**Published:** 2022-03-24

**Authors:** Hjatolah Najafi, Ehsan Bahramali, Mostafa Bijan, Azizallah Dehghan, Mehdi Amirkhani, Maryam Balaghi inaloo

**Affiliations:** 1 Department of Health in Disasters and Emergencies, School of Management and Medical Information, Health Human Resources Research Center, University of Medical Sciences, ShirazShiraz, Iran; 2 Noncommunicable Diseases Research Center (NCDRC), Fasa University of Medical Sciences, Fasa, Iran; 3 Department of Medical Surgical Nursing, Fasa University of Medical Sciences, 81936-13119 Fasa, Iran

**Keywords:** Myocardial infarction, Emergency medical services, Pre-hospital transport, Out-of-hospital acute care

## Abstract

**Background:**

In the medical management of acute myocardial infarction, the transport of patients and primary care provided by emergency medical technicians (EMTs) and paramedics are effective in reducing the mortality and disabilities. Therefore, the present study aimed to compare the outcomes of emergency medical services (EMS) vs. non-EMS transport of patients with ST-segment elevation myocardial infarction (STEMI) in southern Iran.

**Methods:**

This is an analytical, cross-sectional study. The study population consisted of the individuals registered in Fasa Registry on Acute Myocardial Infarction (FaRMI) in the south of Iran. 2244 patients with STEMI were included in the study. Statistical analyses were performed using Chi-Square test and independent t-test at a significance level of *P* < 0.05 in SPSS 22.

**Results:**

Out of the 2244 patients with STEMI, 1552 (69.16%) were male and 672 patients (29.94%) were female. 934(41.62%) patients used EMS transport to the hospital, while 1310 (58.37%) patients used non-EMS transport to the hospital. A total of 169 patients with STEMI (7.26%) expired (out-of-hospital cardiac arrest); of them, 113 (66.86%) patients did not use EMS transport to the hospital. Successful cardiopulmonary resuscitation (CPR) was performed on 52 patients who used EMS transport. 27 patients also received an effective DC shock due to ventricular fibrillation (VF). Of the total number of patients, 49 had a stroke; among them, 37(75.51%) patients did not use EMS transport.

**Conclusion:**

In the present study, the death rate in patients with acute myocardial infarction who used EMS transport was lower than those who used non-EMS transport. The health system managers and policymakers in the healthcare systems are recommended to take the necessary measures to increase public health awareness and knowledge about the use of EMS and consequently reduce the death rate and complications of acute myocardial infarction.

## Introduction

The prevalence of cardiovascular diseases (CVD), which is the leading cause of death, has become one of the most threatening challenges to the healthcare system in most societies [1]. Acute myocardial infarction with ST-segment elevation (STEMI) is one of the most important cardiovascular diseases, with the highest rates of hospitalization and mortality [2]. The costs of diagnosis, treatment, and the re-admission of patients with myocardial infarction impose a heavy economic burden on patients and the healthcare system [3]. According to the World Health Organization (WHO), 50% of deaths in developed countries and 30% in developing countries are caused by acute myocardial infarction [4].

Myocardial infarction, commonly known as a heart attack, is the first cause of death in Iran (47% of deaths) [5]. Several studies have shown that although it can have disastrous consequences, including death, stroke and heart failure, its proper management, effective and timely clinical practices, and the appropriate and rapid transport of patients to medical centers play a critical role in reducing the complications of the disease and the rate of mortality [6, 7]. Half of the patients with acute myocardial infarction die if they are left untreated in the early hours; this occurs since prompt diagnosis and timely treatment, such as primary angioplasty and administration of thrombolytic drugs which restore the blood flow to a blocked artery, have been successful and have reduced the severity of the consequences and complications [8]. The time of initiation of the treatment plays a crucial role in managing and treating acute myocardial infarction; for instance, the European Society of Cardiology recommended that primary angioplasty should be performed in a period less than 120 min after the onset of myocardial infarction symptoms [9].

There is a direct correlation between the time of initiation of treatment and that of the patient’s transport to medical centers. Żurowska-Wolak et al. [10] stated that the shorter the transport time of the patients with acute myocardial infarction, the better results the therapeutic interventions would have. Consequently, the mortality and severe side effects caused by myocardial ischemia will be reduced [10]. Alrawashdeh et al. [11] indicated that delays in the emergency medical services (EMS) in patients with acute myocardial infarction, especially those with haemodynamic instability, increased the total time to reperfusion and were directly related to 30-day mortality after STEMI. However, the delay is sometimes due to the Emergency Medical Treatment before the transport of the patient to the hospital, which leads to better treatment outcomes in the hospital [11].

In addition to the time of transporting the patients, the EMS in the ambulance during the transport can also reduce the consequences of myocardial infarction [12]. Patients often use various methods to go to the medical centers, affecting the time of access to treatment and emergency medical services during the transport. Pathan et al. conducted a study in the United States and Qatar in 2012–2014 and compared the transport of patients with acute myocardial infarction by air and ground ambulances. They found that although both ambulances provide EMS by trained paramedics in the transport of the patients, air ambulances (i.e., a specially equipped helicopter) can better observe the standards of intervention time for opening the blocked arteries and reducing the rate of mortality [13]. Jollis et al. [14] showed that patients with acute myocardial infarction who used EMS transport to medical centers had better results in therapeutic interventions and had a shorter length of stay at the hospitals than those who used non-EMS transport.

## Background in Iran

EMS services in Iran are publicly founded and are free for all members of the community. Pre-hospital emergency services include urban and road emergency bases that are distributed throughout the country. EMS technicians who have a Master’s degree, bachelor’s or Master’s degree in nursing, emergency technician or anesthesia nurse, provide services in the pre-hospital emergency system and have the necessary skills to interpret ECG and diagnose acute myocardial infarction. If a heart attack is diagnosed, contact the supervisor of the destination hospital and inform him/her to activate the myocardial infarction code; also the Interventional Cardiologist should be informed to be prepared for a possible PPCI.

In the medical management of acute myocardial infarction, the time of EMS and therapeutic interventions are the most influential factors affecting the consequences of myocardial infarction, Accordingly, the proper transport of patients and primary care provided by emergency medical technicians and paramedics are effective in achieving the desired therapeutic objectives. Patients choose different methods to go to the medical centers; for example, some people may use EMS transport, while others may choose other methods, such as private or public transport. On the one hand, the method of transport of patients with acute myocardial infarction may affect the consequences of the disease. On the other hand, despite the great importance of the issue, no study has been conducted on the issue in Iran; therefore, the present study aimed to compare the consequences of EMS vs. Non-EMS transport of patients with ST-segment elevation myocardial infarction in southern Iran.

## Methods

**Fig. 1:**
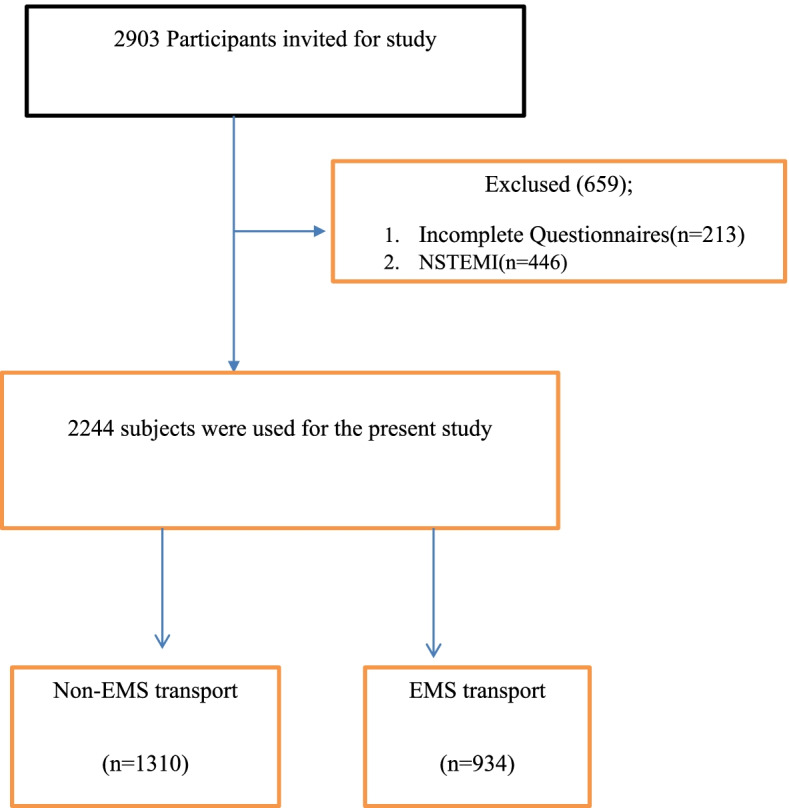
Participant recruitment flow diagram

This is an analytical, cross-sectional study. Data were collected from 2015 to 2020. The study population consisted of the individuals registered in Fasa Registry on Acute Myocardial Infarction (FaRMI) in the south of Iran. This center was founded in 2015 to register patients with acute myocardial infarction in Fasa, Fars province, Southern Iran [15]. This registers all patients admitted to intensive care units (ICUs) or coronary care units (CCUs) with a diagnosis of myocardial infarction. Patients’ personal health information is recorded in this center by two well-trained nurses under the supervision of a cardiologist. The participants in the present study were selected according to consensus sampling. Accordingly, a total of 2,244 participants were found to be as eligible and then invited to participate in this study (Fig. 1).

In order to gather the data, a questionnaire consisting of demographic information, medical histories, risk factors for heart disease, symptoms, time of symptom onset, the method of transport of patients to a medical center, care and treatment provided before, during, and after hospitalization, the tests results and complications which occurred during the transport to the hospital, and the length of stay. We also extracted necessary information about patients who died during transportation using EMS and forensic medicine documents and asked their family members to sign the consent forms.

The inclusion criteria in the present study were all patients with STEMI and willingness to participate in the study, while the exclusion criteria were the patients with incomplete questionnaire information and those with no STEMI.

Statistical analyses were performed using Chi-Square test and independent t-test in SPSS software (version 22.0). The *p*-value less than 0.05 was considered statistically significant.

## Ethical considerations

This paper was extracted from a research project with the ethical code (IR.FUMS.REC.1399.036) in Fasa University of Medical Sciences, Fasa, Iran. The present study was conducted in terms of the principles of the revised Declaration of Helsinki, a statement of ethical principles that directs physicians and other participants in medical research involving human subjects. All the subjects were informed about the objectives of the study, the voluntary nature of their participation, the data collection methods, and confidentiality and anonymity of the information. Subsequently, they were asked to sign the informed consent form if they were willing to participate. The participants were notified that they are free to withdraw from the study at any time.

## Results

Out of the 2244 patients with STEMI, 1552 (69.16%) were male and 672 (29.14%) were female. The mean age of the participants was 55 ± 4.5 years. Table 1 illustrates other demographic characteristics. "In total 169 patients experienced an out-of-hospital cardiac arrest (OHCA), with the majority (66.86%) occurring in the cohort of patients that did not utilise EMS transport to hospital. Survival outcomes from OHCA were significantly different depending on mode of transport to hospital, with 92% (52/56) of patients transported by EMS surviving in comparison with 1.77% (2/113) in the non-EMS cohort (*p* < 0.001)."

**Table 1:** Clinical and demographic characteristics of the study population

Variable	All		EMS		Non-EMS		*P*-value
	N	%	N	%	N	%	
Gender							
Male	1552	69.16	546	58.45	815	62.21	0.975
Female	672	29.94	388	41.54	495	37.88	
Marital status							
Marred	1278	56.95	624	48.82	654	51.17	0.141
Single	669	29.81	312	46.63	357	53.36	
Divorced	198	8.82	119	60.10	79	39.89	
other	99	4.41	68	68.68	31	31.31	
Smoking history							
Never	1427	63.59	611	42.81	816	57.18	0.702
Ex- Smoker	162	7.21	82	50.61	80	49.38	
Current smoker	655	29.18	346	52.82	309	47.17	
Opium abuse							
Never	1526	68	676	44.29	850	55.70	0.354
Ex-consumer	119	5.30	65	54.62	54	45.37	
Current consumer	599	26.39	299	49.91	300	50.08	
Water pipe							
Never	2155	96	981	45.52	1174	54.47	0.141
Ex-consumer	29	1.29	13	44.82	16	55.17	
Current consumer	60	2.67	28	46.66	32	53.33	
Comorbidities							
hyperlipidemia	627	27.94	259	41.30	368	58.69	0.517
Diabetes mellitus	560	24.95	227	40.53	333	59.46	0.432
hypertension	1040	46.34	449	43.17	591	56.82	0.891
Presenting symptom							
Cold sweating	1192	52.1	581	55.0	611	49.0	0.271
Epigastric pain	378	16.05	147	14.1	231	18.0	0.147
Chest pain	1952	85	924	88.0	1028	82.0	˂0.001
Shoulder pain	712	31.0	325	31.0	387	31.0	˂0.001
Jaw pain	163	7.0	75	7.0	88	7.1	˂0.001
ECG change (Dysrhythmia)	74	3.0	25	2.0	49	3.0	.038
Back pain	426	18.0	210	20.0	216	17.0	.092
Syncope	31	1.0	20	1.0	11	0.0	0. 34
Diaphoresis	679	29.0	349	33.0	330	26.0	0.17
Breathlessness	838	36	420	40.0	418	33.0	.001

Chi square test

Successful cardiopulmonary resuscitation (CPR) was performed on 52 patients who used EMS transport. 27 patients also received an effective DC shock due to ventricular fibrillation. Of the total number of patients, 49 had a stroke, of whom, 37(75.15%) patients did not use EMS transport. The average time from the onset of symptoms to arrival in the hospital was 59.45 ± 24.73 min by ambulance and 48.75 ± 56.63 min by other vehicles. Table 2 shows the compares the outcomes of EMS vs. non-EMS transport of patients with STEMI.

**Table 2:** Compares the outcomes of EMS vs. non-EMS transport of patients with STEMI

Outcomes	EMS		Non-EMS		*P*-value
	N	%	N	%	
Death	56	33.93	113	66.86	˂0.001
PCI	557	59.63	465	35.49	˂0.001
CABG	257	27.51	194	14.80	˂0.001
Complete Heart Block	24	43.63	31	56.36	˂0.001
Stroke(cerebrovascular accident)	37	75.51	12	24.48	˂0.001
Successful cardiopulmonary resuscitation (CPR)	52	5.56	2	0.15	˂0.001

Independent sample t test

## Discussion

Prompt diagnosis of myocardial infarction in patients and timely medical interventions play a vital role in reducing mortality, side effects, and disabilities [16]. The condition of patients with acute myocardial infarction is unpredictable, and their life may be at risk due to fatal dysrhythmia at any moment [17]. Therefore, timely clinical practices of emergency service technicians play a very influential role in reducing the rate of mortality and complications of the disease [18]. Alrawashdeh et al. [11] conducted a study in Australia and Canada. They showed that patients with acute myocardial infarction who used EMS transport to the hospital showed better results in primary percutaneous coronary intervention (PPCI) and lower out-of-hospital cardiac arrest rates [11]. Varcoe et al. [19] showed that patients with acute myocardial infarction, who used EMS transport to the hospital in the first minutes, had satisfactory treatment outcomes. Rodrı´guez-Leor, et al. [20] also showed that patients with acute myocardial infarction who used effective EMS transport to the hospital and received proper primary medical care had a lower mortality rate in the ambulance and hospital. This study also showed that the consequences of PPCI in patients with acute myocardial infarction who used EMS transport showed better results than those who used non-EMS transport [20]. According to clinical guidelines, the shorter the access time to interventional treatments, including PPCI in patients with acute myocardial infarction, and the shorter the EMS transport time of the patients to the hospital, the better treatment outcomes [21].

In this study, 1244 patients with acute myocardial infarction (55.43%) used non-EMS transport to the hospital. Also, 169 patients with acute myocardial infarction (7.26%) expired; of them, 113 (5.03%) patients did not use EMS transport to the hospital. Successful cardiopulmonary resuscitations (CPRs) were performed on 52 patients who used EMS transport. 27 patients also received an effective DC shock due to ventricular fibrillation. The results showed that the death rate in patients with acute myocardial infarction who used EMS transport was lower than those who used non-EMS transport. Therefore, it is necessary to provide the required public education about the EMS transport of patients, particularly those with myocardial infarction, by holding culture-building programs. In this regard, Ghasemi et al. [22] showed that one of the most influential factors which reduce the mortality rate in patients with acute myocardial infarction is performing cardiopulmonary resuscitation by emergency service technicians. They illustrated that the lack of knowledge of patients’ companions about how to deal with patients with myocardial infarction and cardiac arrest and not using pre-hospital emergency services in these emergencies increase the rate of mortality [22]. The results of the study of Schultz et al. [23] show that the high survival rates of patients with STEMI that is complicated by OHCA and the important role EMS plays in providing timely CPR and defibrillation, which is consistent with the findings of the present study.

Based on clinical guidelines and management of acute myocardial infarction, primary care practices such as immobilization of the patient, pain relief, use of oxygen, and use of antiplatelet and anti-coagulation drugs such as aspirin play a vital role in preventing and reducing complications in patients with acute myocardial infarction [24]. Therefore, EMS will be beneficial and effective in achieving the mentioned treatment goals. Suppose patients and companions both use non-EMS transfer of their patients to the medical centers. In that case, the patient will be deprived of receiving primary care provided by emergency medical technicians, which will affect the treatment outcome. Since the most common cause of death of patients with myocardial infarction in the first minutes and hours is cardiac dysrhythmias, especially Ventricular fibrillation (VF), in case these patients use non-EMS transport, their rate of death will increase. The study showed that the average time from the onset of symptom to the hospital arrival was 59.45 ± 24.73 min by ambulance and 48.75 ± 56.63 min by other vehicles; however, this time difference was not significant. It can be noted that emergency medical technicians and paramedics perform the necessary primary care and treatment measures and, in some cases, cardiopulmonary resuscitation, which may increase the survival time; on the other hand, patients who do not use emergency medical services may have cardiac arrest during the transport; it is due to the fact that the patient’s companions do not have the necessary knowledge and clinical skills and cannot take adequate measures; these endanger the patient’s life.

## Strengths of the study

It is the first innovative study in Iran which aimed to compare the consequences of EMS vs. non-EMS transport of a patient with acute ST-segment elevation myocardial infarction (STEMI) based on the databases of the FaRMI center.

## The limitations of the study

In this study, only patients with STEMI were evaluated, and other heart diseases such as angina and non-ST-segment elevation myocardial infarction (NSTEMI) were excluded. Since the prevalence of heart disease varies based on cultural, economic, and social factors and this study was only conducted in southern Iran, it is recommended that this study should be conducted in other parts of Iran and other countries. It is also suggested that longitudinal studies should be conducted in this field in order to examine the consequences in more detail. This study was based on FaRMI. The first phase of this registry does not specify certain death times (Pre-hospital mortality, Hospital mortality, but pre-PCI, Mortality during PCI / Intervention, Mortality post-intervention, ideally at 30 days). The first phase lasted about 5 years and was completed in 2020. The second phase of the registry has already started; recording specific times of death is one of the goals of the second phase of the FaRMI registry, which we hope will be examined in future studies.

## Conclusion

In the present study, the death rate in patients with acute myocardial infarction who used EMS transport was lower than those who used non-EMS transport. Since acute myocardial infarction is affected by the initial measures and the transfer of patients to the medical centers, the health system managers and policymakers in the healthcare systems are recommended to take the necessary measures to increase public health awareness and knowledge about the use of EMS and consequently reduce the death rate and complications of acute myocardial infarction.

## Data Availability

The datasets used and/or analysed during the current study are available from the corresponding author on reasonable request.

## Abbreviations

STEMI: ST-Segment Elevation Myocardial Infarction
EMS: Emergency medical services.
CPR: Cardiopulmonary resuscitation
VF: Ventricular fibrillation.
WHO: World Health Organization
FaRMI: Fasa Registry on Acute Myocardial Infarction
OHCA: Out-of-Hospital Cardiac Arrest
