# Correction: Comparison of the outcomes of EMS vs. Non-EMS transport of patients with ST-segment elevation myocardial infarction (STEMI) in Southern Iran: a population-based study

**DOI:** 10.1186/s12873-022-00639-z

**Published:** 2022-04-29

**Authors:** Hjatolah Najafi, Ehsan Bahramali, Mostafa Bijani, Azizallah Dehghan, Mehdi Amirkhani, Maryam Balaghi inaloo

**Affiliations:** 1 Department of Health in Disasters and Emergencies, School of Management and Medical Information, Health Human Resources Research Center, University of Medical Sciences, Shiraz, Iran; 2 Noncommunicable Diseases Research Center (NCDRC), Fasa University of Medical Sciences, Fasa, Iran; 3 Department of Medical Surgical Nursing, Fasa University of Medical Sciences, Fasa, 81936-13119 Iran

**Correction: BMC Emerg Med 22, 1-7 (2022)**

**https://doi.org/10.1186/s12873-022-00603-x**

The original article [1] contained an error in the spelling of Mostafa Bijani’s name which has since been corrected.
